# “Highlighting” red nuclei by atypical posterior reversible encephalopathy syndrome in a patient with systemic lupus erythematosus

**DOI:** 10.1002/ccr3.2245

**Published:** 2019-06-13

**Authors:** Tetyana Vaysman, Prissilla Xu, Tara Vartanian, Peter Michalak, Kesley Pike, Antonio Liu

**Affiliations:** ^1^ Department of Internal Medicine University of Maryland Cheverly Maryland; ^2^ Department of Pharmacy Adventist Health White Memorial Los Angeles California; ^3^ Department of Neurology Adventist Health White Memorial Los Angeles California; ^4^ Marquette Family Residency Program Marquette Michigan; ^5^ Ross University School of Medicine North Brunswick New Jersey

**Keywords:** PRES, red nuclei, systemic lupus erythematosus

## Abstract

This is a case report in which a patient with SLE had a brainstem variant of PRES, and MRI demonstrated atypical distribution of FLAIR hyperintensity in the thalami and the midbrain sparing the red nuclei bilaterally (Figure 1). This impressive lesion pattern may reveal the disease mechanisms of PRES in patients with SLE.

## 1 INTRODUCTION

**Figure 1:**
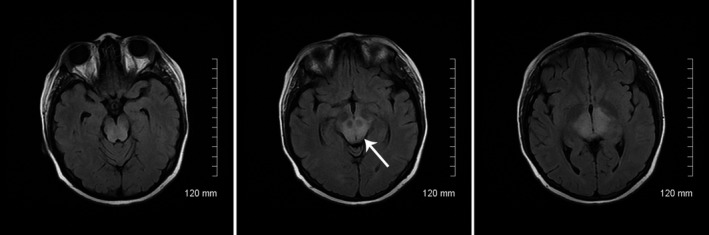
MRI on day 3. MRI T2‐weighted‐FLAIR (fluid‐attenuated inversion recovery) showed midbrain hyperintensity sparing the red nuclei which are hypointense (image in the center)

Posterior reversible encephalopathy syndrome (PRES), a syndrome of grouped findings such as headache, altered mental status, seizures, and vision changes along with posterior leukoencephalopathy on CT or MRI imaging, was first described in 1996 by Hinchey.^1^ Classic PRES usually involves white matter in the posterior part of the brain more susceptible to disruption in autoregulation due to less‐developed sympathetic regulation compared with anterior circulation. During the onset of hypertension, this region is more prone to vasogenic edema.^2^ The syndrome is most commonly encountered with chronic hypertension,^3^ acute kidney injury, chronic kidney disease, eclampsia, pre‐eclampsia, sepsis, immunosuppressive drugs, illicit drugs (cocaine), organ transplantation, collagen vascular disease, autoimmune disorders, and other conditions. PRES has a high potential of rapid reversibility especially when the underlying cause can be effectively addressed. SLE has also been associated with PRES.^4^, ^5^ The literature review of the published studies revealed conflicting results regarding the incidence and prevalence of the CNS involvement in SLE. The frequency of neurological involvement in SLE is ranging from 12% to 95%.^6^ However, the prevalence of PRES was only 0.69% among patients with SLE.^7^ Neuropsychiatric manifestations are common in SLE first and may include, but not limited to SLE cerebritis, stroke, vasculopathy seizures, psychosis, cognitive disorder, headache, migraine, transverse myelitis, optic neuritis, meningitis, and neuropathies. Given the overlapping symptoms, making an accurate diagnosis can be challenging at times, and as the number of PRES reports grows, so do the number of documented of atypical cases.^8^, ^9^ In our presented case, a rapid improvement and resolution of MRI finding with the treatment of hypertension make this much more likely an atypical PRES. Regardless, the “highlighting” of red nuclei (due to presumed sparing) makes an interesting visual pattern (Figure 1).

## 2 CASE PRESENTATION

**Figure 2:**
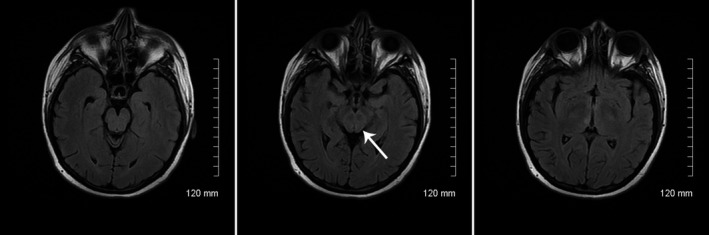
MRI on day 10. MRI T2‐weighted‐FLAIR (fluid‐attenuated inversion recovery) showed near complete resolution of the midbrain hyperintensity when compared to prior imaging (image in the center)

A 22‐year‐old Hispanic female patient with a known diagnosis of systemic lupus erythematosus (SLE) presented to the emergency room complaining of headache, chest pain, muscle pain, and joint pain. SLE was diagnosed roughly 2 years prior, crescentic lupus nephritis type IV biopsy confirmed 5 weeks prior to current presentation. On admission, patient’s renal function was at her baseline and did not fluctuate during the hospitalization course: GFR (glomerular filtration rate) 21, BUN (blood urea nitrogen) 93, and creatinine 3.4. Hemoglobin 9.3 and hematocrit 27.6 were also at her baseline and remained stable following week. Home medications for SLE were azathioprine 25 mg PO once daily, hydroxychloroquine 200 mg PO bid, mycophenolate mofetil 1500 mg PO bid, and prednisone 5 mg PO bid. Five days prior to presentation, she began to experience subjective fever, malaise, and arthralgia, myalgia followed by diffuse throbbing headache without visual deficits at that point. There were no seizures or other neurological deficits. On arrival to the Emergency Department, her chief complaint was severe headache and 1 day of right eye blurry vision. Blood pressure was severely elevated at 197/121. Antihypertensive medications Lasix 80 PO BID, Carvedilol 12.5 PO BID, and Isosorbide Mononitrate 30 PO every morning initiated immediately in the Emergency Department. At that time, CT head without contrast showed no acute findings. Lupus flare was suspected, high dose of Prednisone 50 mg PO daily (in contrast to home dose of Prednisone 5 mg PO daily) was started, and home doses of mycophenolate mofetil and hydroxychloroquine were continued since the admission. Clonidine 0.1 mg q6hr was used for treatment only for the first 2 days. Blood pressure remained high 181/99 mm Hg during the day 1 and 188/116 mm Hg during the day 2. The patient was subsequently worked up for chest pain with elevated Troponin I. VQ scan suggested low probability for pulmonary embolism. Soon after, the patient was thought to have myocardial injury and/or demand ischemia in light of SLE‐associated vasculitis, pericarditis, myocarditis, etc On day 2, neurology was consulted for headache and rapid onset right eye blindness. On examination, closing of the right eye demonstrated 20/25 vision in left eye. However, closing of the left eye resulted in severely decreased visual acuity of the right eye to 20/200. On further examination, despite patient was oriented to time, place, person and situtaion, she appeared drowsy and incoherent on open‐ended question, and there was no active hallucination. Cranial nerve examination beside the vision remained intact; pupils were equal and briskly reactive. Motor examination was nonfocal showing strength of 5 out of 5 at that time. There was no bowel and bladder issue. On day 3, three MRI FLAIR (fluid‐attenuated inversion recovery) sequence demonstrated hyperintensity of this patient’s midbrain and thalami sparing the red nuclei (Figure 1). There was no occipital white matter involvement. With adding nifedipine on day 3 of hospitalization, blood pressure decreased to 160/99 of mm Hg. On day 4, patient began to experience lower extremity weakness with motor strength of 4/5 and ataxic gait without any changes in the reflex (she was never hyperreflexic). She has repeatedly refused spinal fluid analysis. Her blood pressure was 155/100 mm Hg by day 5. All her neurological symptoms were self‐limited and began to improve by day 6. Aggressive blood pressure treatment has brought the BP back down to range SBP (systolic blood pressure) 130‐135 mm Hg and DBP (diastolic blood pressure) 70‐80 mm Hg by day 7. Her eventual discharge blood pressure was 123/78 mm Hg. By day 10 of admission (7 days after original study), MRI FLAIR showed near complete resolution of the midbrain hyperintensity when compared to prior imaging (Figure 2). By then, her neurological symptoms had resolved. SLE serum laboratory results demonstrated C3 and C4 levels were low, 34 mg/dL and <5 mg/dL, respectively. ANA was positive; anti‐dsDNA was positive; and dsDNA QN was elevated at 48 IU/mL. Chromatin was positive, and chromatin QN was elevated at >8 AI. B2Glycoprotein 1 IgM and IgG Ab were normal range, 0 and 2, respectively. Anti‐U1 RNP, anti‐Smith, anti‐Jo‐1, centromere Ab, ribosomal P, anti‐Scl‐70, anti‐SS‐A, and anti‐SS‐B were all negative. Resolution of the patient’s symptoms coincided with MRI improvement and blood pressure control before plasma exchange therapy was concluded. On day 9, TPE (Therapeutic Plasma Exchange), which has never used before, was added by treating rheumatologist to manage SLE acute exacerbation. One of the published studies has concluded that despite long‐term treatment with immunosuppressive medications, some patients still progress to complications and TPE was an effective management with successful outcomes.^10^

## 3 DISCUSSION

Many hypotheses of PRES pathophysiology exist, but the most popular theory attributes to severe hypertension causing interruption to brain autoregulation. It is estimated that around 70% of patients with PRES have hypertension. 3 Failure in autoregulation occurs above a mean arterial blood pressure of 150‐160 mm Hg, and in chronic hypertension, it occurs at higher pressures.^11^ It is very controversial since PRES also occurs in patient with absence of hypertension.^12^ Many scholars support an alternative theory of PRES as the outcome of endothelial damage due to the systemic inflammatory condition associated with sepsis, organ transplantation, eclampsia, and autoimmune diseases.^11^ The study of the “Clinical features and outcomes of posterior reversible encephalopathy syndrome in patients with systemic lupus erythematosus” from 2013 revealed that the prevalence of PRES was 0.69% among patients with SLE. Even though PRES is uncommon in patients with SLE, it is associated with a high mortality rate. The most feared sequelae are ICH (intracranial hemorrhage), rapidly deteriorating renal function, thrombotic microangiopathy, pulmonary hemorrhage, and multiple organ dysfunction syndrome that appeared during 65.4% of observed patients. Hypoalbuminemia and thrombocytopenia were risk factors for PRES‐related ICH.^13^ Another study, conducted in 2017 amount patients with PRES in ICU, revealed that the majority were females with most common symptoms of seizure on presentation following visual disturbances, headaches, and encephalopathy. High blood pressure was observed in over 85% of these patients. It was concluded that prompt recognition and blood pressure control were essential to patients’ survival and recovering rate as well as limitation of residual deficits.^7^ The study of 120 cases with PRES reports that 42% of patients were treated with immunosuppressive medications (cyclophosphamide, tacrolimus, cyclosporine, mycophenolate, bevacizumab, rituximab, vincristine, methotrexate, hydroxychloroquine, 5‐fluorouracil, sirolimus, thalidomide, gemcitabine, paclitaxel, carboplatin, sorafenib, infliximab, and hydroxyurea) and 45% had autoimmune disorders such as thrombotic thrombocytopenic purpura, systemic lupus erythematosus, hypothyroidism, scleroderma, Crohn’s disease, ulcerative colitis, primary sclerosing cholangitis, rheumatoid arthritis, and insulin‐dependent diabetes mellitus.^2^

To elucidate the pathophysiology of the disease, it is necessary to point out that during uncontrolled high blood pressure, the vasoconstriction, as a response of autoregulation, could exacerbate or provoke inflammatory endothelial damage. It leads to hypoxia and, as a result, to the vasogenic edema.^2^ In the autoimmune disease, a microvascular endothelial injury has paved a base to the extravasation of proteins and fluids especially at the sites of ultrafiltration. Circulating immune complexes identified in SLE and other autoimmune diseases have been involved in endothelial cell activation, deposition in the endothelium, and alternating functionality of these cells and contributing greatly to vascular injury by increasing permeability.^14^ This is why aggressive management of hypertension as a factor that further exacerbates endothelial permeability is essential to prevent subsequent damage and to facilitate prompt recovery.^3^ Authors Fugate and colleagues concluded that PRES is highly associated with autoimmune disorders and suggests that endothelial damage is the basis of the pathophysiologic progression of the disease.^7^

To elaborate more on distinctive features of PRES, it would be helpful to mention about Lupus cerebritis, a common feature of SLE, which is an inflammatory response of CNS to increased concentration of cytokines. Lupus cerebritis presents with nonspecific MRI findings and can pose a challenge of deriving a diagnosis which is based on neurological signs, clinical manifestation, and presence of antibodies in CSF (cerebrospinal fluid) and serum.^15^ However, the distinctive feature is imaging, which are not defined in lupus cerebritis in contrast to imaging with pathologic findings and full resolution within days or a week in the PRES.

In this case report, a patient with SLE and hypertension had a course of neurological symptoms consistent with PRES. Typical features in this patient included headache, altered mental status, and MRI lesions that resolved after a few days following closely the lowering of blood pressure. MRI findings in patients with PRES usually reveal bilateral white matter abnormalities in vascular watershed areas in bilateral posterior cerebral hemispheres, with the majority involving the parietal and occipital lobes. Despite clearly defined typical symptoms in our case, some atypical features were a monocular decrease in vision, weakness in the lower extremities, ataxia, and location of the lesion that predominantly affected the midbrain and thalamic area, sparing of typical subcortical white matter. Another unique feature of this case is sparing of the red nuclei with midbrain involvement on MRI imaging (Figure 1).

There are limited published studies on the red nucleus pathology due to complexity of investigating human brain. It is known that red nuclei are located in rostral midbrain and responsible for gross motor movement through the rubrospinal tract.^16^ It is believed that red nucleus inherited its name because of pale red color of the tissue due to high content of hemoglobin and ferritin. On normal MRI, red nuclei present as hypointense structures and it is suggested due to high iron content.^16^ Some published works could shed a light on the discussed topic. Authors who conducted multiple modality MRI study of the healthy human brain speculated that presence of iron in brain tissue could potentially have some effects such as a shift in the local resonance frequency due to iron‐induced magnetic susceptibility and a decrease in the effective T2 relaxation time due to molecular diffusion through the susceptibility induced gradient which results in hypointensity of the red nuclei on MRI imaging.^17^ Pathological MRI findings in our case have revealed hyperintensity in the midbrain and thalami reflecting its involvement and hypointensity in red nuclei which are spared (Figure 1). Considering highly reliable magnetic resonance imaging nowadays, the question what serves as a protective mechanism for sparing on MRI and why red nuclei were not affected by PRES in this case offers as an excellent point for the debates.

## 4 CONCLUSION

We suggest that our case elaborated the significance of an inflammatory state of the SLE and the acute flare of the disease which subsequently triggered the hypertensive crisis and vasogenic response which in turn precipitated posterior reversible encephalopathy syndrome. Few reports of PRES demonstrate involvement of the brainstem. Fewer reports demonstrate isolated midbrain involvement. In this case report, the patient had a PRES presentation with hypertension concurrent with symptoms of a SLE exacerbation. Sparing the red nuclei creates a visually distinctive, perhaps interesting, “highlighting” effect (Figure 1). We hope that this case could raise awareness of atypical presentation and could be useful to utilize a fresh approach in diagnosing, prompt therapeutic interventions and prevention of the neurological decline in SLE patients with PRES symptoms.

## CONFLICT OF INTEREST

The authors declare that they have no conflicts of interest regarding the publication of this paper.

## AUTHOR CONTRIBUTIONS

TV, Department of Internal Medicine, University of Maryland, primary author: assumed responsibility for the publication, making sure that the data are accurate, that all deserving authors have been credited; and responsible for the literature review and final approval, submitting revisions and final version, and communicating with editors. PX, Department of Pharmacy, Adventist Health White Memorial, Los Angeles, co‐author: responsible for data collection and analysis, consulted on patient’s therapeutic pharmacologic regimen, monitoring the progress of the disease, and contributed greatly to the case presentation section in the manuscript. TV, Department of Neurology, Adventist Health White Memorial, Los Angeles, co‐author: provided consults from Neurology Department, contributed to data collection and analysis, provided guidance in manuscript drafting, and contributed greatly to the discussion section of the manuscript. PM, Marquette Family Residency Program, Michigan, co‐author: responsible for data collection and analysis, manuscript drafting, and literature review; and contributed greatly to the discussion and case presentation sections. KP, Ross University School of Medicine, Co‐author: responsible for data collection and analysis, manuscript drafting, and literature review; and contributed greatly to the introduction and case presentation sections. AL, Department of Neurology, Adventist Health White Memorial, Los Angeles, corresponding author: provided consult from Neurology Department and was responsible for supervision, guidance, and final approval of the manuscript; assumed all administrative and executive functions; and ensured the accuracy and integrity of the manuscript were appropriately investigated.
